# Proteomic profiling reveals biomarkers and pathways in type 2 diabetes risk

**DOI:** 10.1172/jci.insight.144392

**Published:** 2021-03-08

**Authors:** Debby Ngo, Mark D. Benson, Jonathan Z. Long, Zsu-Zsu Chen, Ruiqi Wang, Anjali K. Nath, Michelle J. Keyes, Dongxiao Shen, Sumita Sinha, Eric Kuhn, Jordan E. Morningstar, Xu Shi, Bennet D. Peterson, Christopher Chan, Daniel H. Katz, Usman A. Tahir, Laurie A. Farrell, Olle Melander, Jonathan D. Mosley, Steven A. Carr, Ramachandran S. Vasan, Martin G. Larson, J. Gustav Smith, Thomas J. Wang, Qiong Yang, Robert E. Gerszten

**Affiliations:** 1 Cardiovascular Institute,; 2 Division of Pulmonary, Critical Care and Sleep Medicine, and; 3 Division of Cardiovascular Medicine, Beth Israel Deaconess Medical Center (BIDMC), Boston, Massachusetts, USA.; 4 Department of Pathology, Stanford University, Stanford, California, USA.; 5 Division of Endocrinology, Diabetes and Metabolism, BIDMC, Boston, Massachusetts, USA.; 6 Department of Biostatistics, Boston University School of Public Health, Boston, Massachusetts, USA.; 7 Broad Institute of Harvard and MIT, Cambridge, Massachusetts, USA.; 8 Department of Cardiology, Clinical Sciences, Lund University and Skåne University Hospital, Lund, Sweden.; 9 Departments of Medicine and Biomedical Informatics, Vanderbilt University Medical Center, Nashville, Tennessee, USA.; 10 Department of Medicine, Divisions of Preventive Medicine and Cardiology, Boston University School of Medicine, Boston, Massachusetts, USA.; 11 The National Heart, Lung, and Blood Institute’s Framingham Heart Study, Framingham, Massachusetts, USA.; 12 Wallenberg Center for Molecular Medicine and Diabetes Center, Lund University, Lund, Sweden.; 13 Department of Cardiology and Wallenberg Laboratory, Gothenburg University and Sahlgrenska University Hospital, Gothenburg, Sweden.; 14 Department of Medicine, University of Texas, Southwestern Medical Center, Dallas, Texas, USA.

**Keywords:** Endocrinology, Diabetes, Proteomics

## Abstract

Recent advances in proteomic technologies have made high-throughput profiling of low-abundance proteins in large epidemiological cohorts increasingly feasible. We investigated whether aptamer-based proteomic profiling could identify biomarkers associated with future development of type 2 diabetes (T2DM) beyond known risk factors. We identified dozens of markers with highly significant associations with future T2DM across 2 large longitudinal cohorts (*n* = 2839) followed for up to 16 years. We leveraged proteomic, metabolomic, genetic, and clinical data from humans to nominate 1 specific candidate to test for potential causal relationships in model systems. Our studies identified functional effects of aminoacylase 1 (ACY1), a top protein association with future T2DM risk, on amino acid metabolism and insulin homeostasis in vitro and in vivo. Furthermore, a loss-of-function variant associated with circulating levels of the biomarker WAP, Kazal, immunoglobulin, Kunitz, and NTR domain–containing protein 2 (WFIKKN2) was, in turn, associated with fasting glucose, hemoglobin A1c, and HOMA-IR measurements in humans. In addition to identifying potentially novel disease markers and pathways in T2DM, we provide publicly available data to be leveraged for insights about gene function and disease pathogenesis in the context of human metabolism.

## Introduction

The burden of type 2 diabetes (T2DM) continues to increase, with an estimated 700 million cases worldwide by 2045 (1). Metabolic diseases such as diabetes are often present for years before becoming clinically apparent. Given the availability of effective interventions for delaying and/or preventing the onset of T2DM and the increasing burden of the condition worldwide, earlier identification of at-risk individuals is a public health priority (2–4). Furthermore, elucidating novel disease markers may provide additional insights into the pathophysiologic mechanisms relevant to the transition to overt T2DM and identify new molecular pharmacological targets.

While many studies have applied metabolite-profiling technologies toward the identification of T2DM biomarkers (5–9), proteomic analyses in large populations are still lacking. Recent advances in aptamer-based proteomic technologies provide high sample throughput, rendering the profiling of low abundance analytes in epidemiological cohorts far more feasible (10–12). In an initial “proof-of-principle” study, we identified dozens of proteins associated with individual cardiovascular risk factors and the composite Framingham Risk Score in individuals without overt cardiovascular disease (11). Additional studies have applied this technology to identify markers associated with coronary artery disease (13), muscular dystrophy (14), and Alzheimer’s disease (15) in patient cohorts. Proteomic technologies have also been recently applied to diabetes but on a more limited scale (16–21).

Furthermore, the integration of proteomic profiles with genetic analyses has begun to define the genetic architecture of the circulating human proteome (10, 12, 22, 23). Such analyses can identify genetic variants with pleiotropic effects on multiple blood analytes (10, 22). Protein associations with damaging genetic variants may also provide insights into downstream effects on various phenotypic traits and outcomes. Linking circulating proteins to genetic variants that influence their concentrations also offers the opportunity to test for potential causal relationships in model systems. Metabolite profiling studies also provide further information regarding protein function, particularly for circulating enzymes. Here, we leveraged an integrative genomics approach for the discovery of biomarkers for T2DM risk and to implicate potentially causal metabolic pathways in 2 well-phenotyped, population-based cohorts with up to 16 years of clinical follow-up. Through our integrative proteogenomic approach, we highlight the potential functional significance of 2 potentially novel protein associations with incident T2DM; WAP, Kazal, immunoglobulin, Kunitz, and NTR domain–containing protein 2 (WFIKKN2); and aminoacylase 1 (ACY1). We further studied the functional effects of ACY1 in in vitro– and animal-based studies.

## Results

### Proteins associated with risk of T2DM in age- and sex-adjusted models.

**Figure 1:**
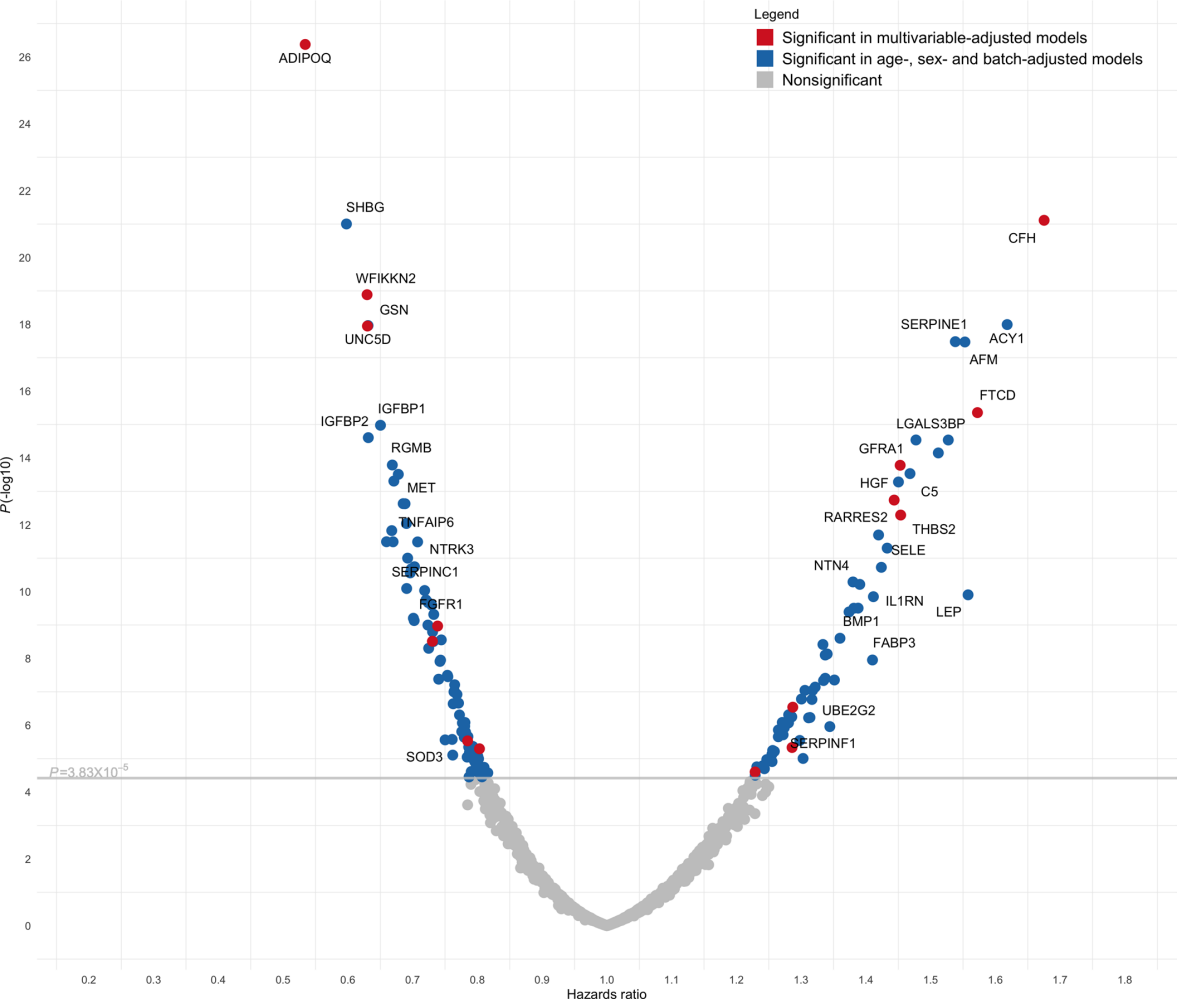
Protein associations with incident T2DM. Volcano plot showing age-, sex-, and batch-adjusted protein associations with incident T2DM in meta-analyses of FHS and MDCS. All colored circles represent Bonferroni significant associations (*P* = 3.83 × 10^–5^) in age-, sex-, and batch-adjusted models. Hazard ratios represent the relative hazard for a 1 SD increment in the transformed and normalized protein level. Red circles represent proteins also found to be significant in multivariable models adjusted for age, sex, batch, BMI, and fasting plasma glucose. Proteins annotated via EntrezGene symbol. See Supplemental Table 1 for protein full name, UniProt, and aptamer sequence IDs.

**Figure 2:**
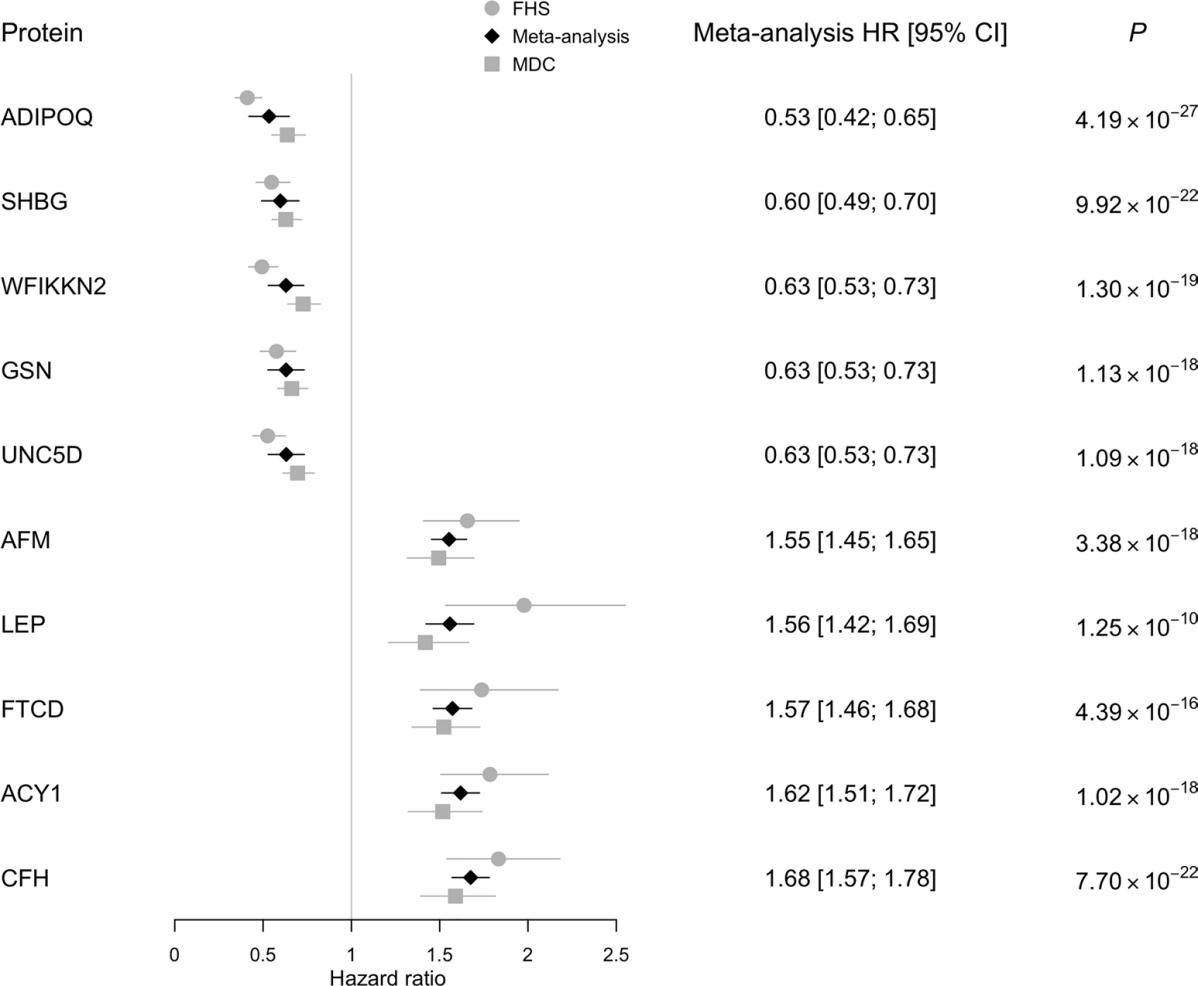
Top protein associations with incident T2DM by cohort level. Top proteins associated with T2DM in age-, sex-, and batch-adjusted models in meta-analyses and by cohort (*P* < 3.83 × 10^–5^). Proteins listed by ascending hazard ratios. Hazard ratios represent the relative hazard for a 1 SD increment in the transformed and normalized protein level. Proteins annotated via EntrezGene symbol. See Supplemental Table 1 for protein full name, UniProt, and aptamer sequence IDs.

Baseline clinical characteristics of the 2839 individuals profiled in both the Framingham Heart Study (FHS) and Malmö Diet and Cancer Study (MDCS) are shown in Table 1. Follow-up time for incidence of T2DM in FHS and MDCS was up to 16 and 15 years, respectively. The overall findings of proteins associated with T2DM risk are provided in the volcano plot in Figure 1. In pooled meta-analyses across FHS and MDCS, we identified 146 proteins that were associated with future risk of T2DM in age-, sex-, and batch-adjusted regression models (*P* < 3.83 × 10^–5^). Representative proteins from these analyses are detailed in Figure 2. We confirmed inverse associations of previously identified markers of T2DM risk, including adiponectin (24) (hazard ratio [HR] 0.53 per 1 SD increment in transformed and normalized protein level; 95% CI, 0.42 to 0.65; *P* = 4.19 × 10^–27^) and sex hormone binding globulin (25) (SHBG, HR 0.60; 95% CI, 0.49 to 0.70; *P* = 9.92 × 10^–22^). However, the majority of the protein associations found were potentially novel in the context of diabetes risk. For example, ACY1, a circulating enzyme that hydrolyzes N-acetyl amino acids into free amino acids (26), had a strong positive association with T2DM risk (HR 1.62 per SD increment; 95% CI, 1.51 to 1.72; *P* = 1.02 × 10^–18^). Additional top findings included Unc-5 netrin receptor D, which had a strong inverse association with T2DM risk (UNC5D; HR 0.63; 95% CI, 0.53 to 0.73; *P* = 1.02 × 10^–18^) and glial cell line–derived neurotrophic factor family receptor α-1, which had a strong positive association (GFRA1; HR 1.45, 95% CI, 1.36 to 1.55; *P* = 1.66 × 10^–14^). Two factors in the alternative complement pathway were also strongly associated with incident disease (27) (Factor H: HR 1.68; 95% CI, 1.57 to 1.78; *P* = 7.70 × 10^–22^; Factor I: HR 1.53; 95% CI, 1.42 to 1.63; *P* = 2.90 × 10^–15^). A list of all age-, sex-, and batch-adjusted protein findings is included in Supplemental Table 1 (supplemental material available online with this article; https://doi.org/10.1172/jci.insight.144392DS1).

### Proteins associated with risk of T2DM in multivariable-adjusted models.

To further evaluate proteins associated with incident T2DM, we performed proportional hazards regression analyses adjusting for established clinical risk factors including age, sex, batch, BMI, and fasting glucose. In pooled meta-analyses across FHS and MDCS, we identified 19 proteins associated with future disease development (*P* < 3.83 × 10^–5^; Figure 1 [red circles], Table 2, and Supplemental Table 2). Many of the protein associations were potentially novel in the context of T2DM risk, including WFIKKN2 (HR 0.77; 95% CI, 0.66 to 0.88; *P* = 6.03 × 10^–6^), a serine protease and metalloprotease that inhibits the biological activity of mature myostatin (28); thrombospondin 2 (THBS2; HR 1.32; 95% CI, 1.21 to 1.42; *P* = 1.95 × 10^–7^), a matrix glycoprotein with anti-angiogenic properties (29); and gelsolin (HR 0.76; 95% CI, 0.65 to 0.86; *P* = 3.35 × 10^–7^), a regulator of actin filament assembly and disassembly (30). Protein associations with incident T2DM were only mildly attenuated even after adjusting for prediabetes (defined as HbA1c 5.7%–6.4% or fasting glucose 100–125 mg/dL) (31) and additional measures of insulin resistance and clinical risk factors (Table 3 and Supplemental Table 3). The median Pearson correlations of the 19 proteins across both cohorts was *r* = 0.10 (range 0.001–0.48). Model discrimination of these 19 proteins was assessed using C-statistic. The addition of these proteins to established clinical and biochemical risk factors (age, sex, BMI, fasting glucose, hypertension, triglycerides, HDL, and batch) improved the C-statistic in both cohorts (FHS: 0.89 [95% CI, 0.86 to 0.92] to 0.92 [95% CI, 0.88 to 0.95]; MDCS: 0.81 [95% CI, 0.79 to 0.83] to 0.85, [95% CI, 0.83 to 0.87]), though the effect was only modest due to very high baseline values.

### Integration of functional genetic variants with T2DM risk proteins.

In order to identify particular proteins that may have functional significance in T2DM risk, we next integrated our proteomics data with genetic information available in the FHS and MDCS cohorts, as well as consortium-based genome-wide association study (GWAS) meta-analyses of T2DM. We performed exome array analyses on 1504 FHS and 1421 MDCS participants to identify rare (minor allele frequency [MAF] 0.1%–1%) and low-frequency (MAF 1.01%–5%) functional genetic variants that are associated with plasma protein levels of the 146 markers associated with age- and sex-adjusted incident T2DM. In meta-analyses of FHS and MDCS exome array data, we identified 21 rare and 48 low-frequency exonic single-nucleotide polymorphisms (SNPs) significantly associated with circulating levels of 44 proteins (*P* ≤ 1.0 × 10^–6^; Supplemental Table 4). Among these findings, 9 rare and 8 low-frequency variants were predicted to be damaging missense, stop gain/loss, or splice-site substitutions located within the coding region of the cognate gene for the measured protein (details including annotation with genotype-phenotype associations with glycemic traits in the Type 2 Diabetes Knowledge Portal and PhenoscannerV2 are in Supplemental Table 5; refs. 32–34). Damaging variants present a naturally occurring genetic perturbation experiment in which effects on blood glucose, HbA1c, and diabetes risk can be assessed on a population basis.

**Figure 3:**
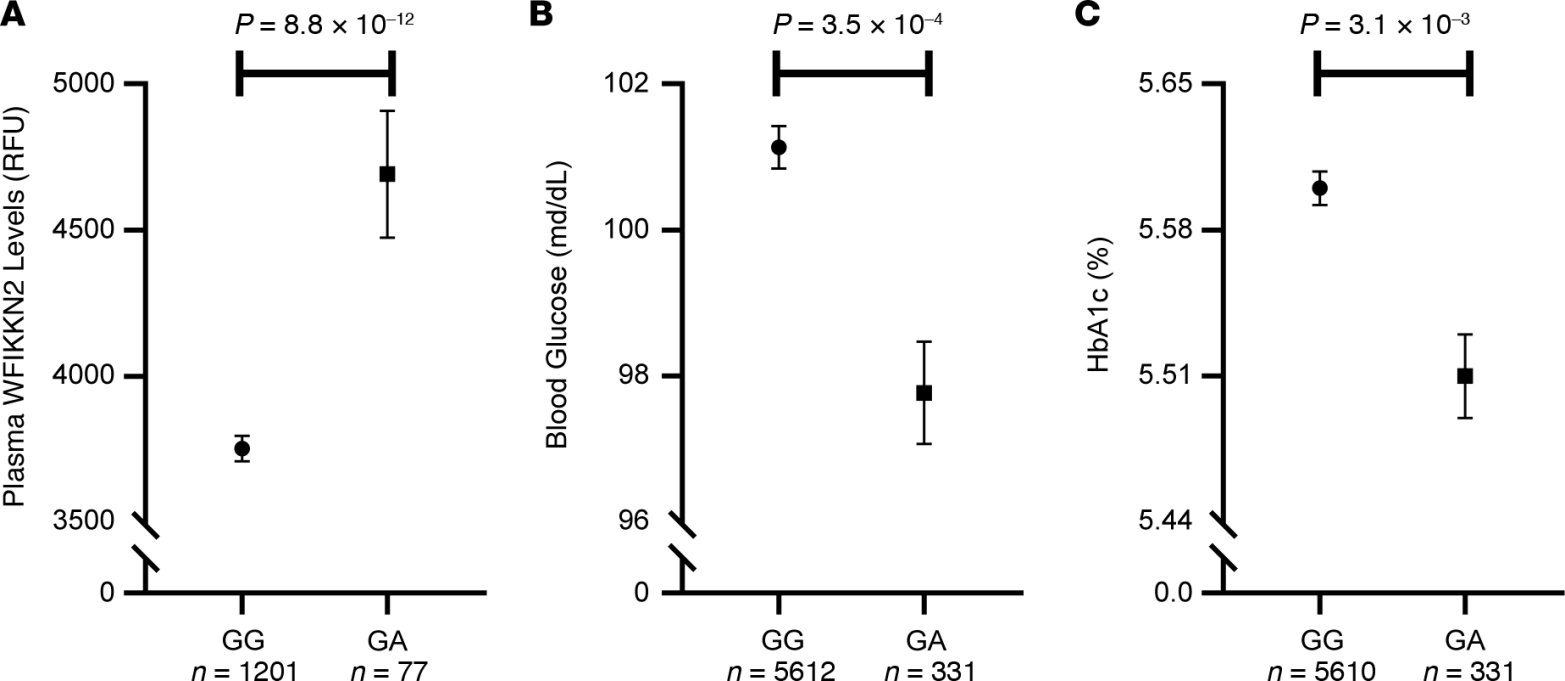
The WFIKKN2 rs35300894 SNP is associated with WFIKKN2 plasma protein levels and glucose homeostasis in FHS participants. Heterozygous carriers of the low-frequency 286G>A, Val96Met missense substitution within the WFIKKN2 gene compared with GG noncarriers in FHS demonstrated significantly (a) higher levels of WFIKKN2 plasma protein levels (mean 4691 ± 217 RFU versus 3754 ± 43 RFU); (b) lower fasting blood glucose (mean 97.8 ± 0.7 mg/dL versus 101.1 ± 0.3 mg/dL); and (c) lower hemoglobin A1c (HbA1c; mean 5.51% ± 0.02% versus 5.60% ± 0.01%). *P* values generated from age- and sex-adjusted regression analyses on natural log-transformed and standardized WFIKKN2, fasting blood glucose, and HbA1c values. RFU, relative fluorescence units.

Examining the 8 low-frequency damaging variants (MAF 1.01%–5%) allowed for improved statistical power to measure the effects on glycemic traits across 5945 genotyped participants of the first-, second-, and third-generation FHS cohorts. Of these, we identified a strong positive association between plasma levels of WFIKKN2 protein and the 286G>A, Val96Met missense substitution in the WFIKKN2 gene (rs35300894; *P* = 8.80 × 10^–12^; β = 0.73; MAF 3.1%). As shown in Figure 3, we found that heterozygous carriers of this variant in FHS (*n* = 331; predicted to have higher plasma levels of WFIKKN2 protein) demonstrated significantly decreased levels of fasting blood glucose when compared with noncarriers (mean glucose GA = 97.8 versus GG = 101.1 mg/dL; β = –0.03; *P* = 3.50 × 10^–4^). Similarly, 286G>A carriers in FHS demonstrated significantly decreased HbA1c levels and HOMA-IR compared with noncarriers (mean HbA1c GA = 5.51 versus GG = 5.60%; β = –0.02; *P* = 3.10 × 10^–3^) and HOMA-IR levels (mean HOMA-IR GA=1.90 versus GG=1.98; β=-0.08; *P* = 0.03). Although there were only 2 FHS participants homozygous for the minor allele (286A), they demonstrated even lower fasting glucose (mean 84.5 mg/dL), HbA1c (mean 5.35%), and HOMA-IR (mean 1.11) levels. Furthermore, this SNP was associated with decrease risk of T2DM after BMI adjustment in the ExTexT2D exome array analysis (odds ratio [OR] 0.95; *P* = 2.98 × 10^–4^; *n* = 228,655) (35), as well as a lower fasting glucose and HbA1c in other exome array association studies (Supplemental Table 5).

We next used Mendelian randomization (MR) methods to test for potential causal association of circulating WFIKKN2 protein with T2DM. We used variants located within the WFIKKN2 gene and associated with circulating WFIKKN2 protein levels in FHS-MDCS meta-analysis (*P* ≤ 0.05) to serve as instrumental variables to estimate the effect of WFIKKN2 plasma protein levels on T2DM, as previously described (36) (see Methods). In addition to the missense rs35300894 variant described above, we identified 3 individual rare and common SNPs (*R^2^* ≤ 0.3) from our meta-analyzed FHS and MDCS exome array (Supplemental Table 4) and GWAS (Supplemental Table 6) results that could be analyzed using publicly available data from the large-scale, consortium-based Diabetes Risk Loci From the Diabetes Genetics Replication and Meta-Analysis (DIAGRAM) study (37) using the inverse variance–weighted MR method (34). These analyses suggest that increased circulating levels of WFIKKN2 decrease T2DM risk with an OR of 0.97 (95% CI, 0.94 to 0.99; *P* = 8.8 × 10^–3^) per unit increase in the rank normal transformed WFIKKN2 levels. These results were consistent with our observed protein findings and support a potential causal association between plasma WFIKKN2 protein levels and clinical T2DM.

To test if circulating WFIKKN2 protein levels and risk of T2DM colocalize to the rs35300894 variant, we applied a Bayesian analysis using the coloc package (38) and publicly available summary data from DIAGRAM (37) (see Methods). These analyses were consistent with our MR findings and demonstrate a posterior probability of 88% that WFIKKN2 plasma levels and risk of T2DM colocalize to the rs35300894 variant.

Our exome array analyses also demonstrated that circulating ACY1 in the FHS Offspring and MDCS cohorts was most significantly associated with a 1057C>T, Arg353Cys missense substitution that has been predicted to disrupt the catalytic subunit of ACY1 (39, 40) (rs121912698, FHS-MDCS meta-analysis; β = –1.8, MAF 0.2%; *P* = 4.50 × 10^–14^). Given the low MAF of this variant, we had limited power to assess variant associations with glucose traits in the FHS and MDCS cohorts. However, a significant association between this loss-of-function variant and decreased fasting blood glucose levels was reported in the FinnMetSeq data set (β = –0.39; *P* = 8.20 × 10^–3^), a larger exome sequencing study of 19,291 Finnish participants (41). These findings were also internally consistent with the increased association of ACY1 and diabetes risk.

### Relation of ACY1 to N-acetylated and free amino acids in humans.

**Figure 4:**
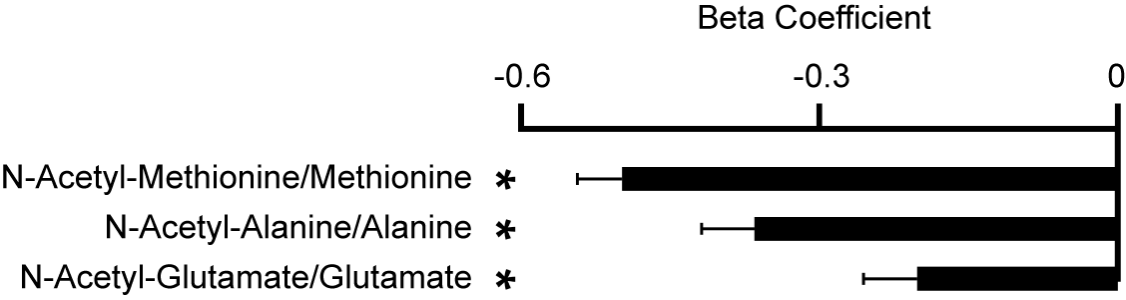
Relation of ACY1 to N-acetylated and free amino acid levels in the MDCS. Shown are the association of circulating ACY1 protein levels with the ratio of N-acetylated amino acid/free amino acid levels (ACY1 substrate/product) in plasma isolated from MDCS participants (*n* = 326). Estimated β-coefficients and *P* values were generated from age- and sex-adjusted regression analyses of plasma ACY1 levels and metabolite levels. Protein and metabolite levels were natural log transformed and then scaled to SD of 1. *P < 0.05.

ACY1 was a top protein associated with age-, sex-, and batch-adjusted incident T2DM and was further highlighted by our genetic analyses. ACY1 hydrolyzes N-acetyl amino acids to generate free amino acids. We and others have previously shown that elevated free amino acid plasma levels appear over a decade before T2DM onset (5, 42), and many studies have documented important roles for multiple free amino acids in insulin secretion and peripheral insulin resistance (43–45). Motivated by the biomarker and genetic associations, as well as our prior work and functional studies related to glucose homeostasis, we first explored the potential role of plasma ACY1 in amino acid metabolism in humans. We analyzed relationships between ACY1 and N-acetylated/free amino acids measured in the MDCS cohort in which both measurements were available (*n* = 326; levels had not been measured in the remainder of MDCS and FHS). As expected, ACY1 levels were inversely associated with the levels of N-acetyl-methionine (β = –0.60; *P* = 1.40 × 10^–33^) and positively associated with free methionine (β = 0.20; *P* = 1.70 × 10^–4^), the canonical substrate and product pair of the enzyme (26). Additional acetylated-to-free amino acid product pairs inversely associated with circulating ACY1 levels included N-acetyl-alanine/alanine (β = –0.37; *P* = 5.20 × 10^–11^) and N-acetyl-glutamate/glutamate (β = –0.20; *P* = 1.20 × 10^–4^; Figure 4). In this smaller MDCS sample with available N-acetyl and free amino acid profiling, N-acetyl/free methionine and N-acetyl/free alanine were also inversely associated with HOMA-IR (N-acetyl-methionine/methionine: β = –0.16, *P* = 7.03 × 10^–8^; N-acetyl-alanine/alanine: β = –0.15, *P* = 8.89 × 10^–6^).

### Effects of ACY1 on amino acid and glucose homeostasis in vitro and in vivo.

**Figure 5:**
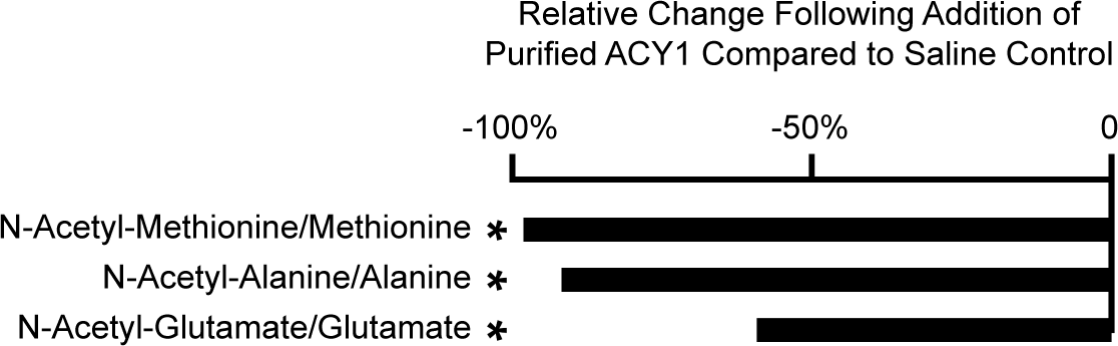
ACY1 modulates N-acetyl and free amino acid levels in isolated human plasma. Relative changes in specific endogenous ACY1 substrate/product ratios (N-acetylated amino acid mean levels/free amino acid mean levels) are shown after human plasma isolated from normal control subjects was treated with purified ACY1 protein (dose = 2.2 nM, *n* = 5) versus a saline negative control (*n* = 5) for 30 minutes at 23°C. *P < 0.05 (unpaired 2-tailed *t* tests).

Next, we tested whether the acute administration of ACY1 was sufficient to alter endogenous amino acid levels in blood in vitro, which has not been previously well characterized. Human plasma was isolated from normal control subjects, and metabolite profiling was performed 30 minutes after the addition of 0.1 mg/mL purified ACY1 or saline control at 23°C (46). We documented decreases in the ratios of multiple ACY1 substrates and products (Figure 5), including N-acetyl-methionine/methionine (–98.1%; *P* = 2.41 × 10^–6^), N-acetyl-alanine/alanine (–91.7%; *P* = 1.19 × 10^–9^), and N-acetyl-glutamate/glutamate (–59.2%; *P* = 5.11 ×10^–10^).

**Figure 6:**
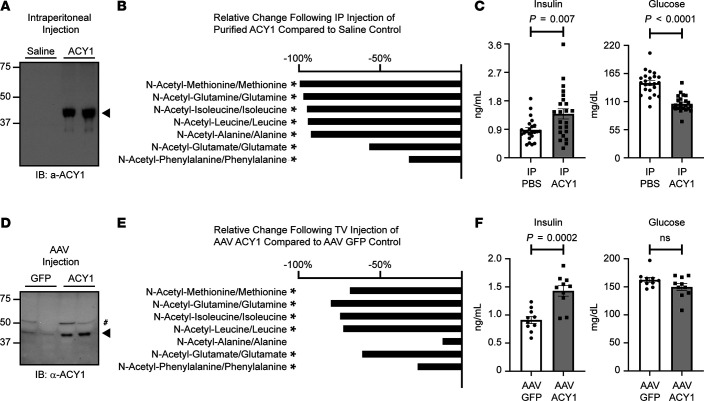
ACY1 modulates amino acid levels and glucose homeostasis in vivo. Mice were injected i.p. with purified ACY1 or saline control. Plasma was collected 6 hours afeter injection. (**A**) Exogenous ACY1 was detected in plasma by immunoblotting with anti-ACY1 antibody (arrowhead). Endogenous ACY1 was also detected with longer exposure times (not shown). (**B**) Significant changes in the ratio of specific endogenous plasma ACY1 substrates/products (N-acetylated amino acid mean levels/free amino acid mean levels) were detected after i.p. injection of ACY1 (100 mg/kg, *n* = 9) compared with saline control (*n* = 9). (**C**) Significant changes in fasting insulin and glucose levels were detected after i.p. injection of ACY1 (*n* = 23) compared with saline control (*n* = 23). Mice were tail vein injected with AAV8 encoding either murine ACY1 or GFP control, and 2-hour fasting plasma was collected by cardiac puncture approximately 40 days after injection. (**D**) Increased levels of ACY1 were detected by immunoblotting with anti-ACY1 antibody in mice injected with AAV-ACY1 compared with AAV-GFP controls (arrowhead, nonspecific band indicated with #). (**E**) Consistent with the i.p. experiments, significant changes in the ratio of specific endogenous plasma ACY1 substrate/product pairs were detected after injection of AAV-ACY1 (*n* = 8) compared with the AAV-GFP control (*n* = 6). (**F**) A significant change in fasting plasma insulin levels was detected after injection of AAV-ACY1 (*n* = 10) compared with AAV-GFP (*n* = 10). *P* values were generated from unpaired 2-tailed *t* tests. *P < 0.05.

Subsequently, we examined whether the administration of ACY1 was sufficient to modulate amino acid levels in a mammalian system. Mice were injected i.p. with saline or purified ACY1 (100 mg/kg), and plasma was collected 6 hours after injection. Western blot analyses performed on plasma collected from ACY1-treated mice demonstrated a band at 46 kDa corresponding to the exogenously administered protein (Figure 6A), while metabolite plasma profiling confirmed functional activity. ACY1 treatment resulted in significant decreases in multiple ACY1 substrate/product plasma ratios (Figure 6B), including N-acetyl-methionine/methionine, N-acetyl-alanine/alanine, and N-acetyl-glutamate/glutamate, consistent with the in vitro findings in human blood.

Given that specific free amino acids generated by ACY1 are potent insulin secretagogues (47–49), we then examined whether acute ACY1 administration also modifies insulin and glucose homeostasis. Following dose-response studies (Supplemental Figure 4), fasting 8-week-old WT C57BL/6 mice maintained on a standard chow diet were injected with saline or purified ACY1 (100 mg/kg i.p.), and plasma samples were collected 6 hours after treatment. We detected a significant 1.6-fold increase in circulating fasting insulin levels following acute ACY1 treatment (1.39 ± 0.16 versus 0.88 ± 0.07 ng/mL; *P* = 6.56 × 10^–3^; *n* = 23), with a concomitant significant decrease in fasting blood glucose levels (105.8 ± 3.26 versus 146.3 ± 4.67 mg/dL; *P* = 7.89 × 10^–9^; *n* = 23; Figure 6C). Of note, administration of N-acetylated amino acid to mice did not elicit decreased fasting glucose levels as observed for the free L-amino acid at early time points (data not shown).

To complement the studies of acute administration of purified ACY1 protein, we generated an adeno-associated viral construct (AAV8) expressing murine ACY1 and repeated glucose and insulin analyses approximately 40 days following tail vein injection with 1 × 10^10^ genome copies of either AAV8 encoding ACY1 or GFP (negative control). As shown in Figure 6D, there was a significant increase in plasma ACY1 protein levels by Western blot in mice treated with AAV8-expressing ACY1 versus GFP. Quantitative Western blot analyses demonstrated an approximate 3.29- ± 0.99-fold increase of plasma ACY1 levels in mice treated with AAV8-expressing ACY1 (*n* = 10) compared with GFP (*n* = 10). Notably, this range of exogenous ACY1 levels was highly comparable with human cohort data, in which we observed a 3.9-fold difference between the lowest and highest quarter of ACY1 plasma levels (mean relative fluorescence units [RFU] 1877 ± 358 versus 7337 ± 4662, respectively) across FHS and MDCS participants. Furthermore, we demonstrated significant decreases in the ratios of multiple plasma ACY1 substrates/products by mass spectrometry (MS) (Figure 6E); these ratios included N-acetyl-methionine/free methionine (–68.5%; *P* = 1.33 × 10^–4^), N-acetyl-glutamine/free glutamine (–80.2%; *P* = 4.31 × 10^–5^), and N-acetyl-isoleucine/free isoleucine (–74.5%; *P* = 1.72 × 10^–5^). Consistent with the acute studies using purified protein, we detected a significant 1.6-fold increase in fasting plasma insulin levels in AAV-ACY1–treated animals as compared with GFP treated controls (1.43 ± 0.09 versus 0.91 ± 0.06 ng/mL; *P* = 2.2 × 10^–4^; Figure 6F). There was a consistent trend toward lower fasting blood glucose levels (AAV-ACY1 [149.8 ± 5.71 mg/dL] versus AAV-GFP [162.4 ± 4.21 mg/dL]; *P* = 0.11; Figure 6F), although this did not reach statistical significance — perhaps due to chronic increases in insulin levels and subsequent compensatory pathways.

**Figure 7:**
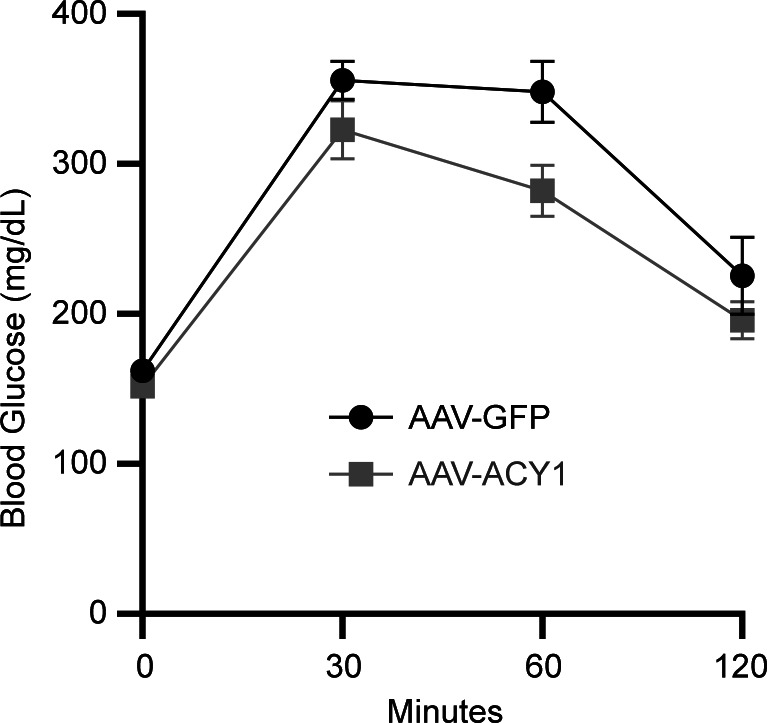
ACY1 modulates glucose tolerance in vivo. High-fat diet–challenged mice were tail vein injected with AAV8 encoding either murine ACY1 or GFP control. I.p. glucose tolerance test was performed approximately 40 days after injection. A significant improvement in glucose clearance at 60 minutes (*P* = 0.02) and whole glucose excursion (as reflected by the glucose AUC; *P* = 0.05) was detected after i.p. glucose loading in mice injected with AAV-ACY1 (*n* = 15) compared with AAV-GFP (*n* = 15). *P* values were generated from unpaired 2-tailed *t* tests. *P < 0.05.

To study the effect of ACY1 in the context of impaired glucose tolerance and early T2DM, we repeated the AAV experiments in C57BL/6J mice that were challenged with a high-fat diet (HFD, 60% energy by fat). As expected, when compared with the AAV-GFP control mice studied above on a normal chow diet, 15-week-old AAV-GFP male mice on a HFD for 9 weeks had significantly higher body weights (35.8 ± 1.27 g versus 31.2 ± 0.56 g; *P* = 0.01) and higher fasting blood insulin levels (1.8 ± 0.33 ng/mL versus 0.91 ± 0.06 ng/mL; *P* = 0.03), with well-compensated, mildly elevated fasting baseline blood glucose levels (163.4 ± 7.45 mg/dL versus 162.4 ± 4.21 mg/dL; *P* = 0.92) and impaired glucose elimination during i.p. glucose tolerance test (IPGTT; AUC 35,542.0 ± 1,890.8 versus 2,0113.1 ± 1,124.04; *P* = 2.2 × 10^–5^). Consistent with our prior findings, we detected higher baseline fasting plasma insulin levels (2.1 ± 0.34 ng/mL versus 1.8 ± 0.33 ng/mL; *P* = 0.63) and lower fasting plasma glucose levels (151.2 ± 8.40 mg/dL versus 163.4 ± 7.45 mg/dL; *P* = 0.29) in 15-week-old AAV-ACY1– versus AAV-GFP–treated mice on a HFD approximately 40 days after tail vein injection. Notably, there was no difference in body weight between AAV-ACY1– versus AAV-GFP–treated mice on HFD (35.8 ± 1.27 g versus 35.6 ± 1.25 g; *P* = 0.77). The difference in baseline insulin levels did not reach statistical significance, perhaps due to the higher baseline fasting insulin levels of these mice compared with the normal-chow diet animals. However, IPGTT analyses revealed a significantly improved glucose clearance at 60 minutes (282.1 ± 16.41 mg/dL versus 348.1 ± 19.54 mg/dL; *P* = 0.02) and whole glucose excursion (AUC 30,531.0 ± 1482.24 versus 35,542.0 ± 1890.82; *P* = 0.05) in mice injected with AAV-ACY1 compared with AAV-GFP controls (Figure 7), highlighting the potential functional significance of ACY1 on insulin and glucose homeostasis.

## Discussion

Using an aptamer-based proteomic profiling platform, we identified 146 plasma proteins with fasting baseline levels that were associated with the future development of T2DM in healthy, nondiabetic individuals up to 15 years prior to disease onset. The proteomic scans confirmed prior knowledge, including the positive associations of adiponectin (24) and the vitamin E binding glycoprotein afamin with T2DM (50), as well the inverse association of SHBG with T2DM (25). Our analyses revealed many potentially novel findings, as well. Proteins highlighted in the age-, sex-, and batch-adjusted models may point to the biological underpinnings of diabetes pathogenesis, including pathways associated with risk factors such as obesity (Supplemental Table 1). Prominent among these findings was the association with a circulating enzyme, ACY1 (*P* = 1.02 × 10^–18^). In addition, those proteins — including WFIKKN2 (*P* = 1.30 × 10^–19^), a serine protease and metalloprotease inhibitor that reduces the biological activity of mature myostatin — that survive rigorous clinical adjustment and Bonferroni adjustment may also prove to be useful clinical biomarkers (28). In multivariable-adjusted models, higher fasting concentrations of 10 proteins were associated with increased T2DM risk; these proteins included GFRA1 and THBS2, which demonstrated greater than 2-fold increased risk when comparing participants in the highest versus lowest quarter of protein concentrations. We also report 9 proteins associated with lower T2DM risk — several that were associated with a nearly 2-fold risk reduction when comparing individuals in the highest versus lowest quarter of protein levels (Table 2 and Supplemental Table 2).

Several studies have used aptamer-based proteomic profiling as a tool for biomarker discovery for specific diseases (11, 13, 15), but few have reported protein associations with incident T2DM after rigorous clinical risk factor adjustment. An important strength of our investigation is the use of 2 large, well-characterized prospective cohorts with diabetes-free participants followed for almost 15 years, enabling us to demonstrate that changes in circulating protein levels can occur well before the diagnosis of diabetes by standard clinical and laboratory measures. All individuals in our study were free of diabetes at baseline sample collection; thus, potential confounding from medical or lifestyle interventions were mitigated, albeit not completely eliminated. Additionally, most of our findings remained significant even upon adjusting for prediabetic state.

Our laboratory investigations focused on ACY1, one of the top circulating enzymes associated with increased risk of future T2DM in age- and sex-adjusted analyses, which was also highlighted by genetic analyses. ACY1 hydrolyzes N-acetyl-L-amino acids to free amino acids (39). We and others have previously demonstrated elevations in circulating free branched chain, aromatic, and other amino acids over a decade before T2DM onset. Furthermore, there is a rich literature describing the acute effects of amino acids as insulin secretagogues and other roles relevant to glucose homeostasis (43–45, 47–49). ACY1 is most highly expressed in metabolically active tissues such as the liver and kidney (51). While ACY1 has been detected in human plasma using liquid chromatography–tandem MS (LC-MS/MS) (52), ELISA (53), and a DNA aptamers (12), the mechanism by which ACY1 enters the circulatory system needs further elucidation. ACY1 does not contain a putative signal peptide, as annotated by standard bioinformatics tools (54, 55). However, recent LC-MS/MS profiling of conditioned medium from primary human hepatocytes and the human liver hepatoma cell line HepG2 identified endogenous ACY1 accumulation in the medium (56). These findings suggest that ACY1 may be secreted by hepatocytes (or other tissues) into human plasma through a nonclassical secretory pathway, although further investigation is needed.

To begin to assess whether ACY1 may act extracellularly on N-acetyl amino acids in humans, we studied the association of ACY1 with classic N-acetyl amino acid substrates and free amino acid products in plasma samples from participants of the MDCS; these species were measured in MDCS but were not measured in FHS previously). We demonstrated that ACY1 levels are indeed inversely associated with N-acetyl amino acid substrates and positively associated with free amino acid products in human plasma (Figure 4). We also found that the addition of purified ACY1 to human plasma is sufficient to catalyze the deacetylation of a range of endogenous N-acetyl amino acids to their free amino acid species (Figure 5). While these studies support the possibility that ACY1 may act extracellularly, further investigation is also required to determine the location of where ACY1 enzymatic activity is occurring to modulate circulating levels of substrate N-acetyl- and product-free amino acid plasma levels in humans.

ACY1 deficiency (OMIM 609924) has been reported as a rare autosomal recessive inborn error of metabolism characterized by the increased urinary excretion of specific N-acetyl amino acids and the childhood onset of severe neurologic abnormalities (40); however, effects on insulin resistance have not been previously described. While prior studies have reported the presence of elevated ACY1 levels in individuals with prevalent and incident T2DM (21), no studies of which we are aware have explored the functional significance of this finding in insulin and glucose homeostasis. Recent murine studies have implicated peptidase M20 domain containing 1, a closely related hydrolase that also regulates N-acyl amino acid levels, in a variety of metabolic phenotypes, including glucose homeostasis (57). Thus, prior work supporting the associations of free amino acids generated by ACY1 with T2DM risk, as well as the functional role of these metabolites on insulin secretion, motivated our studies to test whether ACY1 is sufficient to modulate insulin homeostasis in model systems.

We found that short-term overexpression of ACY1 by i.p. injection or AAV-mediated delivery resulted in increased levels of circulating free amino acids and blood insulin levels (Figure 6). This initial increase in insulin was associated with a modest decrease in fasting blood glucose levels, as might be expected while β cell function is still intact. These findings may be consistent with a model by which increased levels of circulating free amino acids generated by ACY1 lead to increased pancreatic stimulation and secretion of insulin. Intriguingly, while our proteomics and genetic findings in humans demonstrated a highly significant, reproducible, strong positive association between circulating levels of plasma ACY1 and incident T2DM, our short-term studies of ACY1 overexpression in mouse models consistently demonstrated an inverse association between ACY1 and insulin resistance and between ACY1 blood glucose levels. This difference in directionality may be attributable to the chronicity of ACY1 dysregulation. For example, chronic pancreatic hyperstimulation from elevated free amino acids levels generated by ACY1 could eventually lead to β cell exhaustion, reduced β cell mass, and ultimately insulin deficiency and T2DM.

In an effort to capture this potential transition from (a) short-term ACY1 overexpression in murine models being associated with increased amino acid levels and β cell stimulation with hyperinsulinemia to (b) β cell exhaustion with insulin deficiency and T2DM, we repeated the AAV-ACY1 overexpression experiments in a well-established diet-based model of impaired glucose tolerance and early T2DM. We posited that this added stress may accelerate the progression from healthy to impaired β cell function. In 15-week-old male mice that had been maintained on a HFD for 9 weeks, we were not able to observe this transition and, instead, recapitulated the inverse association between ACY1 overexpression and baseline glucose levels and glucose tolerance that we observed in the i.p. and AAV experiments on a normal chow diet. It is likely that longer-term exposures to increased ACY1 levels may be required to study the mechanisms by which ACY1 may ultimately lead to insulin resistance and T2DM in animal models.

Taken together, these studies experimentally validate the roles of ACY1 in glucose and insulin homeostasis that were identified through our functional genomics pipeline and provide proof-of-principle findings that additional targets identified through this approach may also point toward potentially new biological insight. The precise mechanisms by which ACY1 is secreted into plasma, acutely mediates enhanced insulin secretion, and ultimately may lead to T2DM with impaired β cell function and insulin resistance remain to be elucidated and warrant further investigation.

In addition to nominating many potential disease protein biomarkers, we integrated genetic information from exome array and GWAS with proteomic information from the FHS and MDCS, as well as genetic information from large diabetes genetic consortia, to identify proteins that may potentially play a causal role in T2DM. We, along with other investigators, have previously performed GWAS to identify genetic variants associated with circulating levels of proteins measured by the aptamer-based proteomic assays (10, 12, 22, 23). Exome array analyses highlighted damaging, rare, and low-frequency functional variants associated with measured protein levels and altered glycemic traits in carriers when compared with noncarriers. In particular, our exome array results demonstrate that a damaging variant in the coding region of the WFIKKN2 gene is associated with increased levels of circulating WFIKKN2 protein. Although protein-altering variants may have effects on aptamer binding affinity, we have established a pipeline that includes technical validation with MS (11, 58) and/or orthogonal affinity reagents. Supplemental Table 8 summarizes orthogonal data supporting aptamer specificity for WFIKKN2, ACY1, and other proteins reported in our study. The damaging variant in the WFIKKN2 gene is associated with decreased levels of glucose, HbA1c, and HOMA-IR in FHS and decreased risk of T2DM in large consortia data. Our findings suggest that higher measured levels of WFIKKN2 protein are associated with a lower hazard ratio for incident T2DM across FHS and MDCS. The rs35300894 286G>A missense variant is predicted to result in a valine-to-methionine residue change in a highly conserved region of the N-terminus of both isoforms of WFIKKN2. The precise molecular consequence of this substitution is unclear. Whether increased levels of plasma WFIKKN2 associated with this substitution reflect a gain-of-function, or alternatively, a compensatory response to a loss of function of the protein will require future investigation. This multiomics strategy integrating proteomic information with common and rare variant genetic data also highlighted additional protein biomarkers that can be prioritized for future functional studies.

Our study has several limitations. Although the aptamer-based platform applied here provides broad coverage with high throughput, it remains agnostic to changes of proteins not assayed. While FHS participants are closely followed over serial examination to ensure reliable incident diabetes diagnoses, MDCS diabetes cases were identified via registry data. We did not have 2-hour oral glucose tolerance testing (OGTT) results available in either cohort for more precise diabetes diagnosis. We were also unable to differentiate between types 1 and 2 diabetes cases in both cohorts, but based on the prevalence of type 1 diabetes and our study population age, we expect few misclassifications. Use of registry data in MDCS may also introduce the possibility of misclassification of diabetes status; however, we believe such misclassifications would bias us toward the null hypothesis of no association between proteins and diabetes risk. The robustness of our protein findings across both cohorts increases our confidence in the validity of our results. While the data collected from FHS and MDCS are not from a contemporary cohort, given the increased incidence of T2DM over the past 2 decades, we believe the overall importance of our study may be magnified. Storage time and other factors may have contributed to the degradation of some proteins, though this would also bias our analyses toward the null, and we were reassured by the replication of previously reported associations of proteins such as SHBG (25), afamin (50) and adiponectin (24) with incident T2DM. These cohorts were also composed of participants who identified as White. Future proteomic and genetic studies are needed to assess the applicability of our findings in racially diverse and heterogeneous cohorts. Finally, our analyses highlighted proteins for which genetic and/or MS information verified the specificity of the aptamer-protein relationships, though follow-up studies leveraging human genetics, MS, or orthogonal assays will ultimately be necessary for all of the proteins identified in our investigations.

In summary, we have discovered several promising disease markers for T2DM by leveraging a high-throughput, affinity-reagent–based proteomics platform in 2 large epidemiologic cohorts. We then confirmed the functional effects of ACY1, a top finding for incident T2DM, through several experiments in model systems. Furthermore, we present a functional genomic conceptual pipeline integrating proteomic and common and rare variant genomic data from large cohorts and publicly available consortia to highlight additional proteins and pathways that may contribute to disease pathogenesis.

## Methods

### Human cohort study participants

The FHS Offspring cohort was formed in 1971 with the enrollment of 5124 individuals in a community-based longitudinal cohort study (59). Of the 3236 Exam 5 participants (1991–1995) with available plasma samples, 1913 samples had proteomic profiling completed. After excluding individuals with prevalent T2DM or missing clinical data, proteomics data from 1618 participants (177 incident T2DM cases and 1441 noncases) were analyzed in a case-cohort study designed to investigate markers associated with incident T2DM.

The first batch of participants (*n* = 693) was sampled across 899 participants from an incident cardiovascular disease case-cohort study design (5), whereas the second batch of participants was sampled across randomly profiled participants in the cohort (*n* = 925). T2DM was defined as fasting glucose > 126 mg/dL and/or use of insulin or other antidiabetes medications (60). The definition of T2DM was unchanged from baseline through follow-up period.

The MDCS is an epidemiological study of 6103 individuals who were enrolled between 1991 and 1996 as part of a Swedish longitudinal population–based cohort (61). Proteomic profiling was performed on 1661 samples. After excluding individuals with prevalent diabetes and missing clinical data, 1221 participants were included in our incident diabetes case cohort study (272 individuals who developed T2DM and 949 individuals who did not). Similar to FHS, the first batch of MDCS participants (*n* = 614) was sampled across 651 participants from nested case-control studies for incident diabetes and coronary heart disease (CHD), while the second batch of participants was selected from randomly profiled participants across the cohort (*n* = 607). In MDCS, diabetes at baseline was defined as self-report of a physician diagnosis, use of diabetes medication, or fasting glucose ≥ 126 mg/dL. New-onset diabetes diagnosed after the baseline examination until December 2013 (mean follow-up of 13 years) was assessed in subjects free from diabetes at baseline by 3 registers: the Malmö HbA1c registry (MHR), the nationwide Swedish National Diabetes Register (NDR) (62), and the regional Diabetes 2000 register of the Scania region (63). The definition of T2DM was unchanged throughout the study. CHD was defined as new cases of myocardial infarction (MI) and death from CHD in individuals free from history of MI. Baseline clinical characteristics for both cohorts are shown in Table 1.

#### Proteomic analyses in cohort studies.

In both FHS and MDCS, because proteomic data represented relative quantitation and data were collected in batches, protein measures were first log-transformed and standardized to mean = 0 and SD = 1, within respective batches for each cohort. This generated standardized units that allowed proteomic data to be combined across batches. The combined data were then rank normalized for each cohort. The rank normalized data were regressed on Plate ID (i.e., the 96-well plate in which the samples were assayed) to create plate-adjusted residuals, which were used in all analyses in each cohort. Cox proportional hazards regression models were performed adjusting for age, sex, batch, BMI, and fasting glucose in the FHS and MDCS incident T2DM analyses. Fixed effects inverse variance–weighted meta-analyses were conducted to combine the results from the analyses in the FHS and MDCS cohorts. Each of the models for FHS and MDCS were adjusted for the same clinical covariates in secondary analyses. We used a Bonferroni corrected threshold *P* < 3.83 × 10^–5^ to account for statistical tests across all proteins assayed on the platform. All analyses were performed with SAS Software version 9.4 (SAS Institute). Figures were generated with Graph Pad Prism 5 and R version 3.6.3.

#### Proteomic profiling.

The single-stranded DNA aptamer-based SomaScan proteomics platform was applied to FHS and MDCS cohort plasma samples that had been stored at –80°C. FHS samples were collected between 1991 and 1995 and had undergone 2 prior freeze-thaw cycles. MDCS samples were collected between 1991 and 1996 and had never been previously thawed. FHS samples were profiled in 2 batches; the first batch, using Version 1.1, contained 1129 aptamers; the second batch, using Version 1.3, contained 1305 aptamers. The MDCS cohort samples were assayed in 2 batches with Version 1.3. Aptamers that were unique to FHS SomaScan Version 1.1 were not included in our analyses, so only 1062 aptamers in FHS participants from Batch 1 (*n* = 1618) were included in meta-analysis. In SomaScan Version 1.3, used to assay FHS participants in Batch 2, there were 243 new aptamers that were added/replaced on the newer platform. Consequently, protein data for 1305 aptamers were available in FHS Batch 2 participants (*n* = 925) for meta-analyses. Protein data for all 1305 proteins were available for the entire MDCS cohort (*n* = 1,221). In summary, our analyses evaluated 1305 aptamers targeting 1305 unique proteins in each cohort. FHS median intraassay CV was < 4% and median interassay CV was < 7% across batches. MDCS median intra- and interassay CVs were < 4% and < 5%, respectively, across batches. CVs for proteins associated with incident diabetes are listed in Supplemental Table 7. All assays were performed using SomaScan reagents according to the manufacturer’s detailed protocol (64).

#### Metabolomic profiling.

Metabolites were measured in mouse plasma using LC-MS methods previously published by our group (65). In this method, 10 μL aliquots of plasma were deproteinized using a 75:25 methanol/acetonitrile solution with isotopically labeled internal standards (10 μM valine D8 and 10 μM phenylalanine D8). Following vortexing (5 seconds) and centrifugation (21,913*g*, 20 minutes, 4°C), the supernatants were transferred to glass autosampler vials with 300 μL inserts for analysis. LC-MS data were acquired using a HILIC Chromatography on a 2.1 × 150 × 3.5 μm Atlantis HILIC column (Waters). The chromatography system included an Agilent 1200 series LC with a CTC PAL Autosampler. Mobile phase A consisted of 10 mM ammonium formate in water with 0.1% formic acid, and mobile phase B consisted of 100% acetonitrile, with 0.1% formic acid (all components were Optima LC-MS grade; Thermo Fisher Scientific). The injection volume was 10 μL. Initial mobile phase conditions were 5% mobile phase A and 95% mobile phase B, followed by a constant gradient to 60% mobile phase A and 40% mobile phase B over 10 minutes. The column was then reequilibrated to initial mobile phase conditions over 20 minutes. The chromatography system was coupled to an Applied Biosciences/Sciex 4000 QTRAP mass spectrometer with an electrospray ionization source run in negative mode. MRM transitions were optimized for each species on the LC-MS system using Analyst Software (Sciex). LC-MS data were quantified using Agilent MassHunter Quantitative Analysis software. All metabolite peaks were manually reviewed for peak quality in a blinded manner. Pooled plasma was interspersed throughout the run at regular intervals in order to monitor temporal drift in MS performance.

#### ACY1 in vitro studies.

Purified porcine ACY1 (MilliporeSigma, catalog A3010) was dissolved in calcium- and magnesium-free Dulbecco’s PBS (Thermo Fisher Scientific, catalog 14190094) and added to a final concentration of 0.1 mg/mL to 10 μL of human pooled plasma isolated from normal control subjects. Following incubation at 23°C for 30 minutes, the reaction was stopped by adding 75:25 methanol/acetonitrile extraction buffer and subjected to metabolic profiling, as described above.

### Animal studies

#### Murine ACY1 i.p. injection experiments.

For i.p. injection experiments, 8-week-old male C57BL/6J mice (the Jackson Laboratory; stock no. 000664) were injected with purified porcine ACY1 (Sigma-Aldrich, catalog A3010) using a dose of 100 mg/kg body weight or saline in a total volume of 10 μL/gram body weight. Following a 6-hour fast, blood glucose was measured from a tail vein sample using a Contour glucometer (Bayer). Blood was then collected for additional studies by cardiac puncture.

#### Murine AAV-ACY1 experiments.

For AAV injection experiments, 8-week-old male C57BL/6J mice were injected via tail vein with 1 × 10^10^ virus/mouse diluted in saline in a total volume of 100 μL/mouse. Approximately 40 days after tail vein injection, mice were fasted for 2 hours. Blood glucose was measured from a tail vein sample using a Contour glucometer (Bayer). For glucose tolerance tests, mice were fasted for 2 hours. Blood glucose was measured from tail vein samples at 30-minute intervals following i.p. injection of 2.0 mg glucose/gram body weight in 10 μL saline/gram body weight. Blood was then collected for additional studies by cardiac puncture. For high-fat diet studies, mice were maintained on rodent diet with 60 kcal% fat (Research Diets Inc., catalog D12492i) starting at 6 weeks of age.

#### AAV production.

pCMV6-ENTRY plasmid containing a full-length mouse ACY-1-myc-ddk insert was purchased (NM_025371, OriGene Technologies) and subcloned into pENN.AAV8.CB7.CI.WPRE.rBG (Penn Vector Core, Philadelphia, Pennsylvania, USA) using the EcoRV/XhoI sites. AAV-GFP virus (AAV8.CB7.CI.eGFP.WPRE.rBG) was purchased from Penn Vector Core. AAV-ACY1 virus was generated by the Boston Children’s Hospital Viral Core (Boston, Massachusetts, USA) using the pENN.AAV8-ACY1 plasmid.

#### ELISAs.

Insulin ELISA (Crystal Chem, catalog 90080) was used to measure insulin levels from plasma samples according to the manufacturer’s instructions.

#### Western blot.

Plasma samples (0.25 μL) were mixed with Laemmli buffer, separated on a 10% Mini-PROTEAN TGX polyacrylamide gels (Bio-Rad) using sample and running buffers containing SDS, transferred to nitrocellulose membranes, and immunoblotted using anti-ACY1 (Abcam, catalog AB133635) antibodies.

#### Metabolomic profiling.

Metabolites were measured in mouse plasma using the same LC-MS methods described above for the human studies and as previously reported (65).

### Data availability

Aptamer-based proteomic profiling, genome-wide genotyping, and exome array genotyping results for all proteins measured in FHS have been deposited in the database of Genotypes and Phenotypes (https://www.ncbi.nlm.nih.gov/gap) and for MDCS have been deposited in the institutional repository (https://www.malmo-kohorter.lu.se/malmo-kost-cancer-och-malmo-forebyggande-medicin). All other results and analytic methods are available within the manuscript. Details of all commercially available study materials are included in Supplemental Methods. Noncommercial study materials will be made available to other researchers for the purposes of reproducing the results or replication of the procedure, as respective IRB and Material Transfer Agreements permit.

### Statistics

#### Genome-wide genotyping and imputation in FHS.

Genome-wide genotyping methods for the FHS have been described previously (66). Briefly, genotyping was conducted using the Affymetrix mapping array and the Affymetrix 50K gene-focused molecular inversion probes supplementary array. Genotypes were called using Chiam (http://www.stats.ox.ac.uk/~marchini/software/gwas/chiamo.html). We used the 1000 Genomes Phase I version 3 (August 2012) reference panel to perform imputation using a hidden Markov model implemented in MACH (version 1.0.16) (67) for all SNPs passing the following criteria: call rate ≥ 97%, *P* for the Hardy-Weinberg test statistic (pHWE) ≥ 1 × 10^–6^, Mishap *P* ≥ 1 × 10^–9^, Mendel errors ≤ 100, and MAF ≥ 1%.

#### Exome array genotyping in FHS.

Genotyping of the FHS was performed as previously described (68). Genotyping was performed using the Illumina Infinium HumanExome BeadChip (version 1.0). Genotype calling and quality control was performed centrally using all 62,266 samples from participating studies in the CHARGE Consortium (Cohorts for Heart and Aging Research in Genomic Epidemiology) (69). To be included, nonsynonymous, stop-altering, and splice variants needed to be observed ≥ 2 times in ≥ 2 data sets. The array additionally included tags for previously described GWAS hits, ancestry informative markers, random synonymous SNPs, mitochondrial SNPs, and HLA tags (http://genome.sph.umich.edu/wiki/Exome_Chip_Design). In sum, > 240,000 variants were included on the exome array. Of these variants, 103,634 were polymorphic in the FHS samples, which had protein levels measured, and a further subset of 86,567 variants were nonsynonymous, nonsense, or located in a splice site and had a MAF ≤ 5%. A variant was considered damaging if it was a stop gain/loss, splice-altering, or missense variant and was predicted to be damaging by 2 of the 4 algorithms in the database of human nonsynonymous SNPs and their functional predictions (dbNSFP) (Mutation Taster, Polymorphism Phenotyping version 2 [Polyphen 2 HDIV], sorting intolerant from tolerant [SIFT], likelihood ratio test).

#### Genotyping in MDCS.

Genotyping of MDCS participants was performed with the Illumina HumanOmniExpressExome BeadChip version 1.0 or 1.1 and the iScan system (Illumina). This array includes a specific exome part (exome array) of 244,194 primarily exonic markers and also 706,924 markers for coverage of common genome-wide variation as previous described (70). All genotypes were called using the Autocall algorithm. Per-individual QC filters included a call rate > 95% and an inbreeding coefficient of −0.2 to 0.2 to control for excess of heterozygosity. Up to second-degree relatives — identified by estimated identity by descent sharing, subjects mismatched in sex check, and population outliers based on the first 2 principal components — were excluded. Variants with a call rate < 95%, or with Hardy–Weinberg equilibrium *P* < 10^–6^, were excluded. Imputation was performed using Impute 2 with the 1000 Genomes Integrated Phase I release version 3 all populations (ancestry) panels.

#### Genetic association analysis.

Because of skewed distributions of most protein levels, genetic association analyses were conducted using batch-specific rank normal transformed residual values of protein levels adjusting for plate numbers. The association of genetic variants and protein levels was tested using linear mixed-effects models to accommodate pedigree structure in FHS and linear model in MDCS under an additive genetic model, adjusted for age, sex, and first 10 principal components for population admixture (71). GWAS analyses were performed using the R GWAF package (72), and exome array single-variant analyses were performed using the R seqMeta package for FHS and PLINK version 1.07 (http://pngu.mgh.harvard.edu/purcell/plink/) for MDCS. Separate analyses were performed for samples in each batch; then, the results were meta-analyzed using the inverse variance–weighted method for each cohort. The cohort-specific results were then combined using inverse variance–weighted meta-analyses.

#### MR.

To test whether the association between circulating WFIKKN2 and T2DM was consistent with a causal relationship, we performed MR analyses using the genetic association results of WFIKKN2 and T2DM. Associated genetic variants (*r^2^* ≤ 0.3, meta-analysis *P* < 0.05, imputation quality > 0.3) in GWAS (MAF > 0.01) and exome array analyses of circulating WFIKKN2 in FHS and MDCS served as instrumental variables to estimate the effect of WFIKKN2 plasma protein levels on diabetes status. In order to limit the pleiotropic effects of the genetic variants, we used only variants that mapped to the WFIKKN2 gene. Association results of T2DM were obtained from existing 1000G GWAS and exome array data from the DIAGRAM consortium. MR analyses were performed using the inverse variance–weighted method implemented in R package MR (36). Briefly, the effect size of the WFIKKN2 on T2DM was estimated as the ratio of the 2 association coefficients of a genetic variant on T2DM and WFIKKN2 plasma level. Then, the estimated effects of WFIKKN2 on T2DM across all included genetic variants were summarized into a single estimate using the inverse variance–weighted meta-analysis method that also accounts for linkage disequilibrium among genetic variants.

#### Colocalization.

To test whether plasma WFIKKN2 levels and T2DM risk colocalize to the rs35300894 variant, we applied the coloc package (38) using GWAS and exome array data from FHS and MDCS, as well as publicly available summary data from DIAGRAM. Evidence of colocalization was evaluated using the posterior probability for hypothesis 4 (PP4) that WFIKKN2 plasma levels and T2DM shared the same variant rs35300894. Given our focused study on the rs35300894 variant, we set a prior probability that rs35300894 was associated with both WFIKKN2 plasma levels and T2DM of P12 = 1 × 10^–4^.

### Study approval

The human study protocols were approved by the IRBs of Boston University Medical Center, BIDMC, and Lund University, Sweden. All participants provided written informed consent. All animal experiments were approved by the IACUC at BIDMC. All methods were carried out in accordance with the regulations and guidelines of the Animal Welfare Act and the American Association for Accreditation of Laboratory Animal Care.

## Author contributions

REG, DN, and MDB conceived the study, designed the experiments, analyzed and interpreted the data, and wrote the manuscript. JZL designed the AAV constructs for the animal studies. QY and RW performed analyses and interpreted genetic data. MJK performed the statistical analyses and interpreted proteomic data. AKN, JEM, and XS performed metabolomics experiments. ZZC, BDP, DHK, and UAT contributed to data analysis and manuscript generation. AKN, DS, and CC performed animal studies experiments. DS, SS, and LAF performed proteomic assays. MGL and RSV performed experimental design, performed statistical analyses, and contributed to manuscript revision. EK and SAC designed, performed, and analyzed the MS-based proteomic experiments and contributed to manuscript writing. JGS and OM performed replication analyses in the MDCS cohort and contributed to manuscript revision. JDM, TJW, and QY interpreted data and contributed to the manuscript revision. DN and MDB are co–first authors; the order of authors was decided because DN was involved in the earlier phases of study conception.

## Supplementary Material

Supplemental data

Supplemental Tables 1-9

## Figures and Tables

**Table 1:**
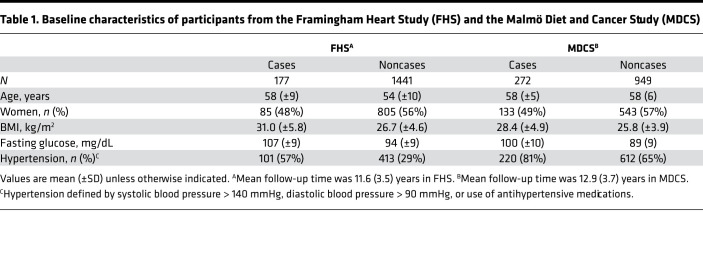
Baseline characteristics of participants from the Framingham Heart Study (FHS) and the Malmö Diet and Cancer Study (MDCS)

**Table 2:**
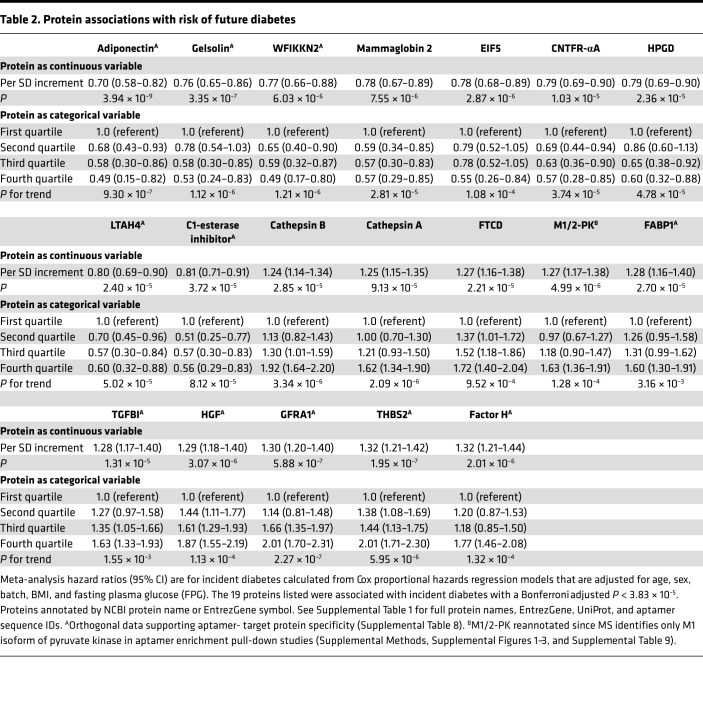
Protein associations with risk of future diabetes

**Table 3:**
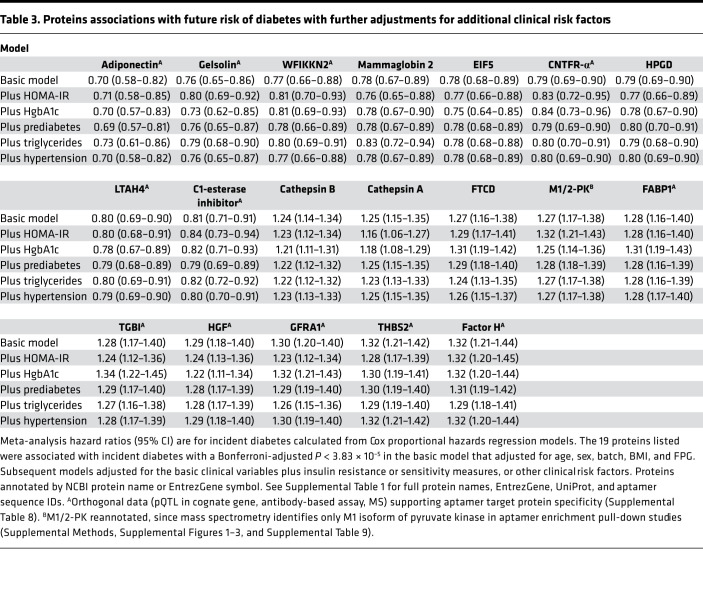
Proteins associations with future risk of diabetes with further adjustments for additional clinical risk factors

## Version 1 02/16/2021

In-Press Preview

## Version 2 03/08/2021

Electronic publication
