# Supplementary material for: Proteomic profiling reveals biomarkers and pathways in type 2 diabetes risk
Source: JCI Insight. 2021 Mar 8;6(5):e144392. doi: 10.1172/jci.insight.144392 (PMC8021115; doi:10.1172/jci.insight.144392)
Supplement: Supplemental data [file jciinsight-6-144392-s289.pdf]

Supplemental Data

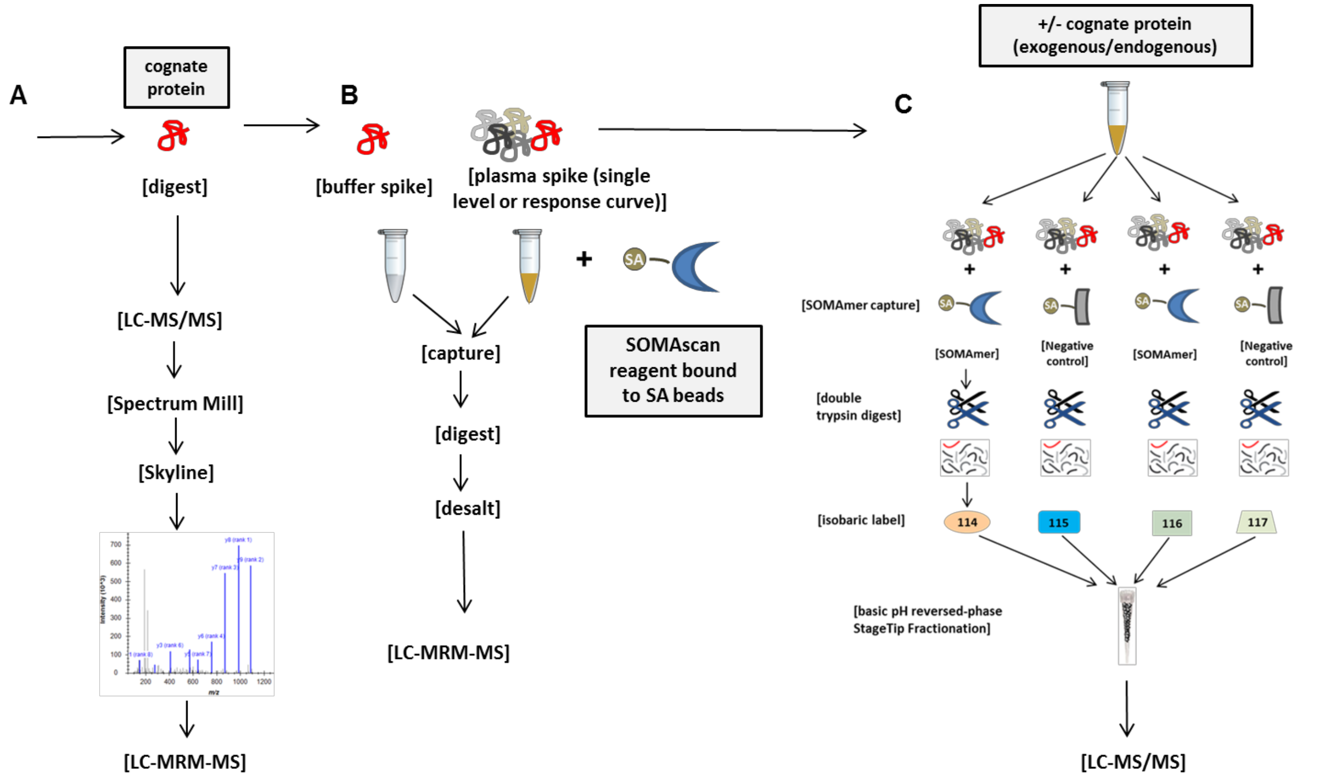

Supplemental Figure 1. MS workflows for selecting peptides and evaluating aptamer affinity using plasma and cognate proteins

Digestion and LC-MS/MS analysis of cognate protein used to select the aptamer to identify the best peptides for targeted MS analysis. **B.** Evaluation of aptamer affinity in buffer and plasma using LC-MRM-MS to quantify the relative abundance of peptides from the cognate protein after aptamer enrichment. **C.** Replicate evaluations of specificity of aptamer using iTRAQ workflow and a negative control aptamer. 500 fmol of cognate protein was added to plasma from a healthy donor pool and enriched in duplicate using the selected aptamer or a negative control aptamer, i.e., an aptamer that has not been iteratively selected against any protein. Each sample was independently labeled with a unique isobaric chemical tag, then the samples were combined and analyzed by LC-MS/MS. Clinical case and control samples were analyzed using the same iTRAQ LC-MS/MS same workflow.

A.

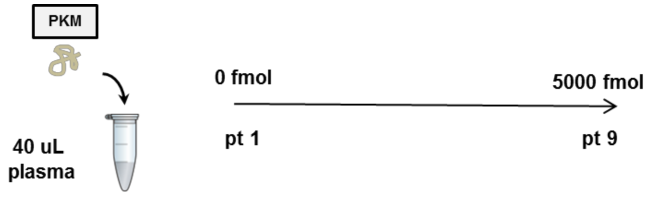

B.

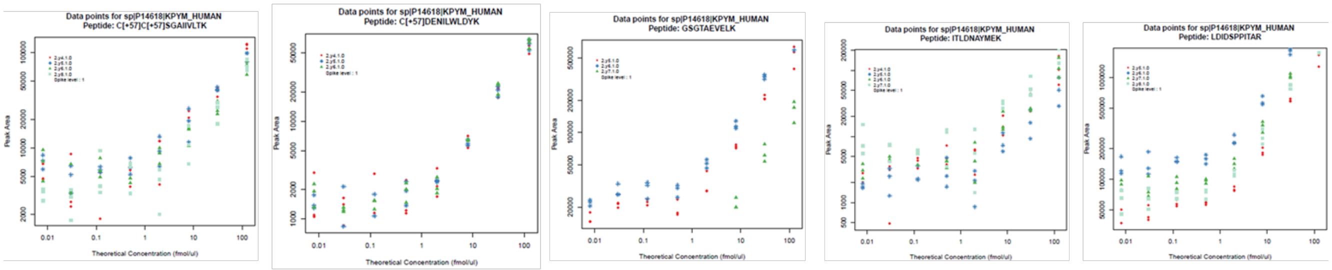

C.

peptide	transition.id	slope	y-intercept	rsquare	LOD (fmol/uL)	LOQ (fmol/uL)
APIIAVTR	2.y5.1.0	109353	345272	0.9416	1.80	5.40
CCSGAIIVLTK	2.y4.1.0	1321	5413	0.9849	6.23	18.68
GSGTAEVELK	2.y6.1.0	9422	25998	0.9322	2.65	7.94
ITLDNAYMEK	2.y7.1.0	2602	5513	0.8601	11.35	34.06
LDIDSPPITAR	2.y6.1.0	46360	143728	0.9706	2.49	7.46

Supplemental Figure 2. Plasma response curves for 5 PKM peptides analyzed by LC-MRM-MS

A. Response curves were prepared by 4-fold serial dilution after adding 5000 fmol of PKM protein (recombinant protein against which the aptamer was derived) into 40 uL of healthy plasma. Each sample was enriched using the PKM aptamer, digested into peptides then analyzed by targeted MS for PKM peptides previously identified by LC-MS/MS. **B.** Results were plotted for peak area for each transition against the theoretical concentration using QuaSAR. **C.** Limits of detection (LOD) and quantification (LOQ) were determined by the method of blank [1] using the peak area of the single best transition for each peptide.

```
PKM1      1 MSKPHSEAGTAFIQTQQLHAAMADTFLEHMCRLDIDSPPITARNTGIICTIGPASRSVETLKEMIKSGMNVARLNFSHGTHEYHAETIKNVRTATESFAS   100
PKM2        MSKPHSEAGTAFIQTQQLHAAMADTFLEHMCRLDIDSPP_____________________________________________________________

PKM1    101 DPILYRPVAVALDTKGPEIRTGLIKGSGTAEVELKKGATLKITLDNAYMEKCDENILWLDYKNICKVVEVGSKIYVDDGLISLQVKQKGADFLVTEVENG   200
PKM2        ____________________________________________________________________________________________________

PKM1    201 GSLGSKKGVNLPGAAVDLPAVSEKDIQDLKFGVEQDVDMVFASFIRKASDVHEVRKVLGEKGKNIKIISKIENHEGVRRFDEILEASDGIMVARGDLGIE   300
PKM2        ____SKKGVNLPGAAVDLPAVSEKDIQDLKFGVEQDVDMVFASFIRKASDVHEVRKVLGEKGKNIKIISKIENHEGVRRFDEILEASDGIMVARGDLGIE

PKM1    301 IPAEKVFLAQKMMIGRCNRAGKPVICATQMLESMIKKPRPTRAEGSDVANAVLDGADCIMLSGETAKGDYPLEAVRMQHLIAREAEAAIYHLQLFEELRR   400
PKM2        IPAEKVFLAQKMMIGRCNRAGKPVICATQMLESMIKKPRPTRAEGSDVANAVLDGADCIMLSGETAKGDYPLEAVRMQHLIAREAEAAMFHRKLFEELVR

PKM1    401 LAPITSDPTEATAVGAVEASFKCCSGAIIVLTKSGRSAHQVARYRPRAPIIAVTRNPQTARQAHLYRGIFPVLCKDPVQEAWAEDVDLRVNFAMNVGKAR   500
PKM2        ASSHSTDLMEAMAMGSVEASYKCLAAALIVLTESGRSAHQVARYRPRAPIIAVTRNPQTARQAHLYRGIFPVLCKDPVQEAWAEDVDLRVNFAMNVGKAR

PKM1    501 GFFKKGDVVIVLTGWRPGSGFTNTMRVVPVP                                                                       531
PKM2        GFFKKGDVVIVLTGWRPGSGFTNTMRVVPVP
```

LEGEND			
Description			No. of peptides ID'd (incl. miscleavages)
Peptide sequenced by LC-MS/MS from SOMAmer pulldown of protein standard spiked into plasma	Red font	**LDIDSPPITAR**	35
Peptides unique to PKM2	Blue font	**LDIDSPPSK**	0

Supplemental Figure 3. PKM peptides identified by MS after aptamer enrichment

Alignment of PKM isoform 1 and 2 with the peptides identified by LC-MS/MS after aptamer enrichment highlighted in red. None of the five peptides unique to PKM isoform 2 was identified. In these overlapping regions, the only peptide identified was unique to PKM 1, suggesting that the PKM1 isoform was enriched using the aptamer.

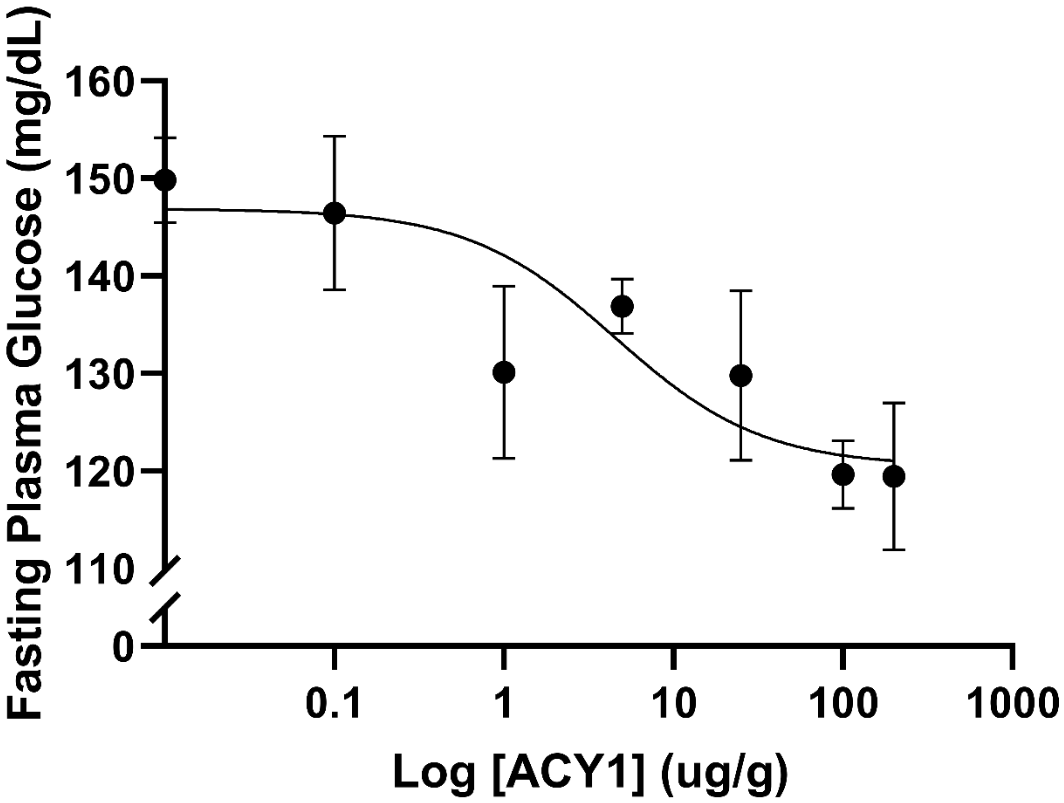

Supplemental Figure 4. Dose response of IP injection of purified ACY-1 protein on fasting plasma glucose in C57/Bl6 mice. Six-hour fasting plasma glucose levels are shown six hours after IP injection of increasing doses of purified ACY-1 protein in C57/Bl6 mice (n=7).

Supplemental Tables 1- 9.

Please see Supplemental Excel Data Tables.

Supplemental Methods

Clinical Definitions for outcomes and traits

FHS: Family history of diabetes was self-reported and defined as type 2 diabetes in one or both natural parents. Prediabetes was defined as fasting blood glucose 100-125mg/dL. Body mass index (BMI) was calculated by dividing weight in kilograms by the height in meters squared. Hypertension was defined as blood pressure ≥ 140/90 mmHg or use of anti-hypertensive therapies. Glucose, insulin, and HbA1c measurements were obtained using previously described methods[2]. In summary fasting plasma glucose levels were measured using hexokinase reagent kits with reported intra-assay coefficient of variation (CV) <3%. HbA1c was measured using high-performance liquid-chromatography after an overnight dialysis. The assay was standardized against the glycosylated hemoglobin assays used in the Diabetes Control and Complications Trials with reported mean (SD) values of 5.22% (0.6%) among subjects that had normal glucose tolerance with inter- and intra-assay CVs <2.5%. Fasting insulin levels were measured in EDTA plasma with inter and intra-assay CVs ranging from 5.0-10.0% with a lower limit of sensitivity at 1.1 μU/mL. HOMA-IR was calculated using the equation (fasting plasma insulin × fasting plasma glucose) / 405. Cholesterol levels including fasting triglycerides levels were measured enzymatically using standardized laboratory approaches.

MDC: Prediabetes was defined as fasting blood glucose 5.6-6.9 mmol/L. BMI was calculated using the same formula as in FHS and hypertension was also defined similar to FHS. Glucose, insulin, and HbA1c measurements were obtained using previously described methods according to standard procedures at the Department of Clinical Chemistry at Skane University Hospital in Malmo or Malmo University Hospital[3]. Triglycerides were measured using routine clinical chemistry methods on Cobas instruments at Roche Diagnostic Inc., Mannheim, Germany. HOMA-IR was calculated using the same equation as FHS after conversion of glucose and insulin values to metric system units.

Methods for MS characterization of PKM aptamer

Aptamer affinity enrichment and digestion– Plasma from a healthy pool (BIOIVT), control or diabetic patients was processed for MS analysis as described previously.[4] In brief, 40 uL was diluted to 100 uL with 60 uL of HEPES buffer and streptavidin beads bound with 10 pmol of activated aptamer (heated to 95C then washed with CAPS buffer) were added and the samples were tumble mixed overnight at room temperature. After incubation and wash with HEPES buffer without Tween-20, the bound material was eluted with 2 volumes of 50 uL of 20 mM NaOH. Eluates were pooled, dried and digested following our Urea protocol.[4]

Preparation of protein response curves – Cognate protein (15 pmol) (protein used during the SELEX aptamer selection process) was added to 40 % plasma (40 uL plasma diluted to 100 uL in HEPES buffer) from a pool of healthy donors (BIOIVT) and serially diluted in 4-fold increments over 8 concentrations as previously described.[5] The diluent, 40 % plasma without exogenous recombinant protein standard added was retained as a blank. One hundred microliters was affinity enriched as described above and analyzed by scheduled LC-MRM-MS as described below.

LC-MRM-MS analysis – Samples were resuspended in 10 uL 3% acetonitrile/5% acetic acid, mixed and centrifuged briefly and transferred to HPLC vials and analyzed on a Quantiva triple quadrupole mass spectrometer equipped with an Easy-nanoLC 1000 and Nanospray Flex source (Thermo). Samples were injected (20% of sample) on a 75 um ID PicoFrit column (New Objective) manually packed to 15 cm with Reprosil-Pur C18 AQ 1.9 um media (Dr. Maisch) and heated to 50 °C. MS source conditions were set to as follows: spray voltage 2000, ion transfer tube temperature 300, sweep gas 0, CID gas 1.5 and source fragmentation voltage 10. Five peptides unique to PKM were scanning for 3 to 4 transition ions per peptide with 0.2 Q1 resolution and 0.7 Q3 resolution scheduled with 8 min window using a cycle time of 1.5 s. Where possible, two charge states and transitions were monitored to increase confidence in peptide detection in absence of heavy labeled standards. Collision energies

were set for each peptide based on its precursor m/z using collision energy equation calibrated for our Quantiva QQQ-MS (see **Supplemental Table 7**). Mobile phases consisted of 3% acetonitrile/0.1% formic acid as solvent A, 90% acetonitrile/0.1% formic acid as solvent B. Flow rate was set to 200 nL/min throughout the gradient, 0% - 6% B in 6 min, 6% - 33% B in 59 min, 33% - 90%B in 4 min with a hold at 90%B for 10 min.

iTRAQ labelling of peptides[6] *and BRP fractionation*[7] *for RAP MS*. Desalted peptides were labelled with iTRAQ434 reagent according to the manufacturer's instructions (AB Sciex). Peptides were dissolved in 30 µl of 50 mM triethylamonium bicarbonate (TEAB) pH 8.5 and labelling reagent was added in 70µl of ethanol. Samples were incubated with labelling reagent for 1 h with agitation, and the reaction was quenched with 5µl of 1 M Tris-HCl pH 7.8. Differentially labelled peptides were subsequently mixed and prepared for BRP fractionation on 50 mg SepPak columns according to the following protocol: cartridges were prepared for desalting by equilibrating with methanol, 50% acetonitrile (ACN), 1% formic acid and 3 washes with 0.1% TFA. Samples were loaded on to the cartridge and washed 3 times with 1% formic acid. A pH switch was performed before the collection of BRP fractions with 5 mM ammonium formate at pH 10. BRP fractions were collected at the following ACN concentrations: 10% ACN in 5 mM ammonium formate; 15% ACN in 5 mM ammonium formate; 20% ACN in 5 mM ammonium formate; 30% ACN in 5 mM ammonium formate; 40% ACN in 5 mM ammonium formate; and 50% ACN in 5 mM ammonium formate.

Analysis of LC-MRM-MS Data. Extracted Ion chromatograms (XIC) of all transition ions were integrated using a Skyline document (Skyline daily version 3.5.0.9319 https://brendanx-uw1.gs.washington.edu/labkey/project/home/software/Skyline/begin.view).[8] After peak verification and integration, peak areas were processed using QuaSAR, (R script available as an external tool in Skyline) to perform regression analysis[5] on the curves and calculate the limits of detection (LOD) and quantification (LOQ) of each peptide for each protein.[9] See **Supplemental Figure 2C** for a summary of aptamer performance.

LC-MS/MS analysis of digested aptamer enriched samples. Samples were resuspended in 10 uL 3% acetonitrile/5% acetic acid, mixed and centrifuged briefly and transferred to HPLC vials and analyzed on a QExactive mass spectrometer (Thermo) equipped with an Easy-nanoLC 1000 and a custom built nanospray source (James A. Hill Instrument Services). Samples were injected (20% of sample) on a 75 um ID PicoFrit column (New Objective) manually packed to 20 cm with Reprosil-Pur C18 AQ 1.9 um media (Dr. Maisch) and heated to 50 °C. MS source conditions were set as follows: spray voltage 2000, capillary temperature 250, S-lens RF level 50. A single Orbitrap MS scan from 300 to 1800 m/z at a resolution of 70,000 with AGC set at 3e6 was followed by up to 12 MS/MS scans at a resolution of 17,500 with AGC set at 5e4. MS/MS spectra were collected with normalized collision energy of 25 and isolation width of 2.5 amu. Dynamic exclusion was set to 20 s and peptide match was set to preferred. Mobile phases consisted of 3% acetonitrile/0.1% formic acid as solvent A, 90% acetonitrile/0.1% formic acid as solvent B. Flow rate was set to 200 nL/min throughout the gradient, 2% - 6% B in 1 min, 6% - 30% B in 84 min, 30% - 60%B, 60%-90%B in 1 min with a hold at 90%B for 5 min.

Analysis of LC-MS/MS Data – MS data were analyzed using Spectrum Mill MS Proteomics Workbench software Rev B.04.01.142 (Agilent Technologies, Santa Clara, CA). Similar MS/MS spectra acquired on the same precursor m/z within +/- 60 sec were merged. MS/MS spectra were

excluded from searching if they failed the quality filter by not having a sequence tag length > 0 (i.e., minimum of two masses separated by the in-chain mass of an amino acid) or did not have a precursor MH+ in the range of 600-6000. All extracted spectra were searched against a UniProt database containing human reference proteome sequences downloaded from the UniProt web site on October 17, 2014 with redundant sequences removed and a set of common laboratory contaminant proteins (150 sequences) added. Search parameters included: ESI-QEXACTIVE-HCD-v2 scoring, parent and fragment mass tolerance of 20 ppm, 40% minimum matched peak intensity and 'trypsin allow P' enzyme specificity up to 4 missed cleavages. Fixed modification was carbamidomethylation at cysteine and variable modifications were acetylation of protein N-termini, oxidized methionine, deamidation of asparagine and pyro-glutamic acid. Database matches were autovalidated at the peptide and protein level in a two-step process with identification FDR estimated by target-decoy-based searches using reversed sequences. Protein-peptide comparison report comprised of all validated peptides summarizing the identified peptides for each sample. Further statistical analysis and plots were performed using R scripts.

Supplemental References

1. Keshishian, H., *et al.* Quantification of cardiovascular biomarkers in patient plasma by targeted mass spectrometry and stable isotope dilution. *Mol Cell Proteomics* **8**, 2339-2349 (2009).
2. Meigs, J.B., *et al.* Hyperinsulinemia, hyperglycemia, and impaired hemostasis: the Framingham Offspring Study. *JAMA* **283**, 221-228 (2000).
3. Magnusson, M., *et al.* Low plasma level of atrial natriuretic peptide predicts development of diabetes: the prospective Malmo Diet and Cancer study. *J Clin Endocrinol Metab* **97**, 638-645 (2012).
4. Ngo, D., *et al.* Aptamer-Based Proteomic Profiling Reveals Novel Candidate Biomarkers and Pathways in Cardiovascular Disease. *Circulation* **134**, 270-285 (2016).
5. Kuhn, E., *et al.* Interlaboratory evaluation of automated, multiplexed peptide immunoaffinity enrichment coupled to multiple reaction monitoring mass spectrometry for quantifying proteins in plasma. *Mol Cell Proteomics* **11**, M111 013854 (2012).
6. Wiese, S., Reidegeld, K.A., Meyer, H.E. & Warscheid, B. Protein labeling by iTRAQ: a new tool for quantitative mass spectrometry in proteome research. *Proteomics* **7**, 340-350 (2007).
7. Munschauer, M., *et al.* The NORAD lncRNA assembles a topoisomerase complex critical for genome stability. *Nature* **561**, 132-136 (2018).
8. MacLean, B., *et al.* Skyline: an open source document editor for creating and analyzing targeted proteomics experiments. *Bioinformatics* **26**, 966-968 (2010).
9. Mani, D.R., Abbatiello, S.E. & Carr, S.A. Statistical characterization of multiple-reaction monitoring mass spectrometry (MRM-MS) assays for quantitative proteomics. *BMC Bioinformatics* **13 Suppl 16**, S9 (2012).
